# POPULATION AGING IN INDIA: A MICROLEVEL ESTIMATE USING GRIDDED POPULATION DATA

**DOI:** 10.1093/geroni/igad104.2329

**Published:** 2023-12-21

**Authors:** Nawaj Sarif, Sruthi Anilkumar Hemalatha

**Affiliations:** International Institute for Population Sciences, Mumbai, Maharashtra, India; International Institute for Population Sciences, Mumbai, Maharashtra, India

## Abstract

Population aging is a significant demographic trend that is transforming societies worldwide. Aging populations pose significant challenges to governments and societies, particularly in developing countries such as India, where a large segment is still impoverished and lacks access to adequate health care and social security systems. A lack of accurate data on older populations has been a significant impediment to developing effective policies to address the needs of this vulnerable group. To address this gap, this study tried to examine the micro-level trends and patterns of population aging in India using gridded population data. The study also attempted to estimate the share of the older population in India for the years 2030 and 2040. The study found that India has undergone a dramatic shift in population aging trends, with large intra-state variability. The share of older populations is predicted to rise considerably over the next two decades, highlighting the urgent need for effective policies to address their needs. The study also revealed that population aging patterns differ across India, suggesting that national and state-level measures alone will not suffice to address the needs of older people. Instead, policies directed at smaller units, such as districts and neighborhoods, are critical to ensuring the well-being of older populations. Furthermore, the study highlights the need for accurate and timely data on older populations in India. Such data can enhance policy decisions and help identify areas of need, enabling governments to develop targeted policies that can improve the well-being of older people.

